# Ultraviolet Photodissociation Spectroscopy of [dAMP–H]^−^ at Low Temperature

**DOI:** 10.1021/acs.jpca.5c06525

**Published:** 2025-12-02

**Authors:** Christian Sprenger, Samuel J. M. White, Miriam Westermeier, Gabriel Schöpfer, Franziska Dahlmann, Uma Namangalam, Salvi Mohandas, Sunil Kumar S, Eric S. Endres, Milan Ončák, Roland Wester

**Affiliations:** † Institut für Ionenphysik und Angewandte Physik, 27255 Universität Innsbruck, Technikerstraße 25, 6020 Innsbruck, Austria; ‡ 443874 Indian Institute of Science Education and Research Tirupati, Department of Physics, Panguru 517619, Andhra Pradesh, India

## Abstract

Nucleotide fragmentation after photoexcitation in the ultraviolet is a potential cause for damage to DNA strands. Consequently, the fragmentation process needs to be explored to understand the stability of nucleotides on a molecular level. Here, we present wavelength-dependent relative photoabsorption cross section measurements of [dAMP–H]^−^ below the photodetachment threshold, which lead to fragmentation along several different channels. Several spectral features are observed in the broad absorption peak in the range of 240 to 270 nm, the resolution of which we attribute to the low temperature of 3 K achieved in our cryogenic 16-pole radiofrequency wire trap. These features likely originate from different Franck–Condon-active vibrational bands in only one or two different conformers. Quantum chemical calculations predict that the spectrum originates from a strong ππ* excitation located at the adenine moiety. Furthermore, the wavelength-dependent yield of the five observed photofragments was studied. This revealed no preferred single photofragment, but showed different trends for different fragments as a function of photon energy. Finally, an absolute photofragmentation cross section of [dAMP–H]^−^ was obtained by comparison with the photodetachment cross section of I^–^.

## Graphical abstract

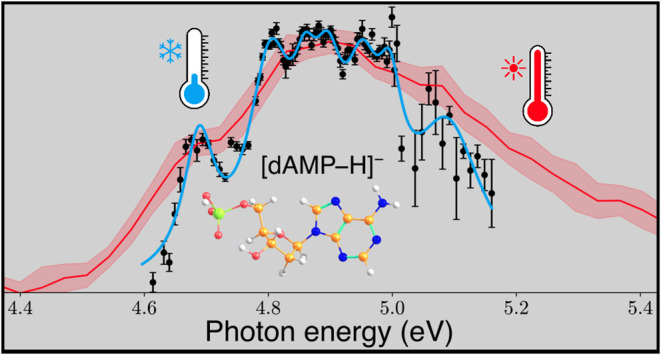

## Introduction

The stability and resilience of deoxyribonucleic acid (DNA), the carrier of hereditary information, against external influences, such as particle collisions or interaction with light, is of fundamental importance for living organisms.^1,2^ It is therefore of great interest to gain an understanding of such interaction processes on the molecular level, i.e. on the level of individual nucleotides. Nucleotides are composed of a phosphate group, a pentose sugar and a nucleobase. While the first two form the backbone of the DNA, the nucleobases encode the genetic information. The nucleobases are strong ultraviolet (UV) absorbers, but they are also exceptionally stable under UV excitation.^3^

Previous works have studied the fragmentation of nucleotides under collision-induced dissociation (CID) to determine activation energies,^4^ fragment branching ratios^5^ and fragmentation pathways.^6,7^ Other works have focused on the interaction of nucleotides with light: among the processes studied were photodetachment,^8,9^ photoionization,^10^ photofragmentation in the UV^11–13^ and infrared^14^ and the effects of the chemical environment on the interaction with light.^15,16^ Theoretical work with a focus on adenine has been done previously.^17–22^

Despite extensive prior work, to our knowledge only one spectroscopic study has examined the photofragmentation of the deprotonated 2’-deoxyadenosine 5′-monophosphate anion ([dAMP–H]^−^) in the gas phase.^23^ This study, by Marcum, Halevi and Weber, was performed on anions at room temperature, which they suggest could mask “sharper features” of the photofragmentation spectrum. However, they already resolved a non-Gaussian shape of the peak near 250 nm.

In this work we report on results of a spectroscopic study of [dAMP–H]^−^ at low temperature, with the aim to obtain more information about its photofragmentation and to benchmark quantum chemical calculations. The spectroscopy was performed in the range of 240 to 270 nm (5.17 to 4.59 eV). The measurements were performed in our cryogenic ion trap setup with a buffer gas temperature of 3 K. Furthermore, the results of wavelength-dependent [dAMP–H]^−^ photofragment yield measurements are shown. These results are discussed in comparison with the results of ref 23. and with quantum chemical calculations of the photoexcitation process. Finally, an absolute photofragmentation cross section of [dAMP–H]^−^ is presented, which was measured by comparing its relative photofragmentation cross section to the photodetachment cross section of I^–^.

## Methods

### Experimental Setup

The experimental setup used for the present measurements has been described previously.^24^ Here we focus on recent improvements of the setup. A drawing of the setup is presented in Figure 1.

**Figure 1:**
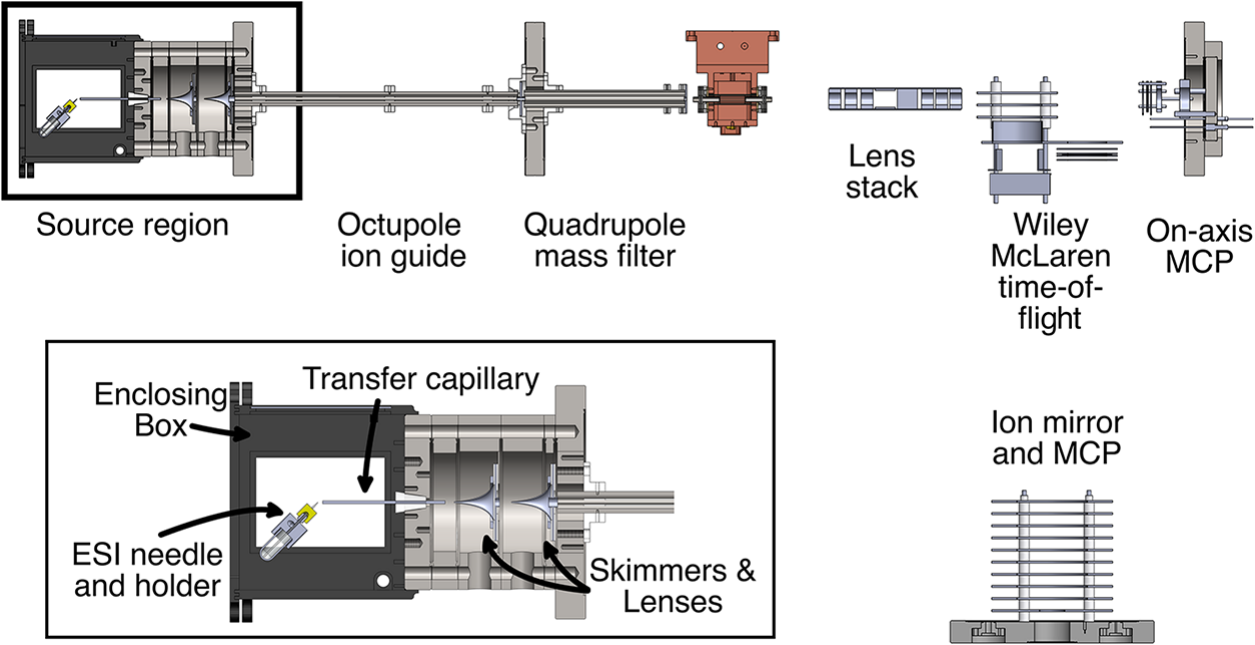
16-pole wire trap setup used for this experiment. A custom-built nano-ESI sprays into a double skimmer source. It guides the ions into an octupole ion guide, which serves as a pretrap. The quadrupole ion filter, with its segmented end-cap, guides the ions into the 16-pole wire trap. After the trap, a lens stack guides the ions into the Wiley–McLaren time-of-flight mass spectrometer. The mirror of the reflectron was not used in the present experiments, instead the ions were detected on a microchannel plate (MCP) behind the mirror.

The ion source has been replaced by a nano-electrospray interface (nano-ESI), which has some advantages over conventional electrospray. First, due to its characteristically low flow rate, the solution takes days of continuous spraying to run out, making long measurement periods possible. Second, the cleaning intervals of the source are longer, since less contamination is introduced into the experiment. Furthermore, the low flow rate and low concentration of a nano-ESI reduces the amount of analyte required for any given measurement. The final advantage of the nano-ESI configuration is its small size and simplicity, which reduces experimental downtime due to maintenance.

The entire interface of the nano-ESI is housed in a protective box, shielding the spray from external influences. This protection is further increased by a constant flow of dry nitrogen into the box. By splitting the flow and passing an adjustable amount of the nitrogen through a wash bottle, control of the humidity inside the box is achieved. Finally, this gas mixture is passed over a heating element, to adjust the temperature around the spray. Careful adjustment of all these parameters gives a greater ion yield and improved stability of the ion source, while reducing disturbing outside influences.

For the present experiments either a 500 μM solution of dAMP in 1:1:1 H_2_O/MeOH/MeCN or a 5 mM solution of NaI in H_2_O with 0.005% by volume acetic acid was used. We observed a reduction in the [dAMP–H]^−^ ion number after the [dAMP–H]^−^ solution was in the heated nano-ESI source for longer than 1 week. The main solution is therefore stored in a refrigerator kept under 10 °C and the solution in the source is replaced once a week. The spray from the nano-ESI is transferred into the vacuum setup via a transfer capillary. After exiting it, two linearly arranged skimmer-lens pairs guide the ions through two differentially pumped stages into the octupole ion guide. A zoom-in view of the source is shown as an inset in Figure 1. The advantage of the double skimmer setup is that it allows the creation of hydrated ion species directly from the source, since the ions experience no high-energy collisions during their transfer into the vacuum chamber, which could break apart ion–water clusters.

After the octupole ion guide, the ions are guided through a single tube lens into the quadrupole mass filter. This was tuned to only transmit ions between about 200 and 360 Da. Excluding heavier ions that are formed in the nano-ESI source, such as deprotonated deoxyadenosine diphosphate anions (410 Da), prevented the unwanted creation of [dAMP–H]^−^ inside the trap by fragmentation of such heavier ions, which could lead to an underestimation of the decay rate of [dAMP–H]^−^. The filtering of the lighter ions prevented unwanted ions from interfering with the mass spectra of the [dAMP–H]^−^ fragments, which all have masses of 195 Da or less. At the exit of the quadrupole, a segmented lens was installed to simultaneously focus and deflect the ion beam into the trap. This enables us to match the center of the ion beam to the opening of the trap, since the trap’s vertical position moves by several hundred micrometers during the cooling-down of the trap.

The trap used for these experiments is a cryogenic 16-pole radiofrequency wire trap described previously.^25^ It is operated at a radiofrequency of 1.9 MHz with a peak-to-peak amplitude of about 600 V and static end-cap voltages of between 1 and 4 V. The trap temperature reaches 3 K during operation. This exceptionally low temperature has previously been proven in our group by achieving multiple He tagging of protonated glycine.^26^

A pulsed OPO laser system (Ekspla NT 242-SH/SF) was employed for the measurements. The pulse rate of this OPO laser system is 1 kHz with a pulse width of ≤ 6 ns and a measured line width of < 5 cm^–1^. For this experiment we used the OPO’s output option that combines the second harmonic generation and sum frequency generation, which can be tuned between 210 and 405 nm.

### Measurement Procedures

A schematic of a typical experimental cycle can be seen in Figure 2. Each cycle begins with loading of the ions already trapped in the octupole guide into the 16-pole ion trap, followed by a period of trapping and exposure to light before the ions are unloaded and analyzed in the mass spectrometer.

**Figure 2:**
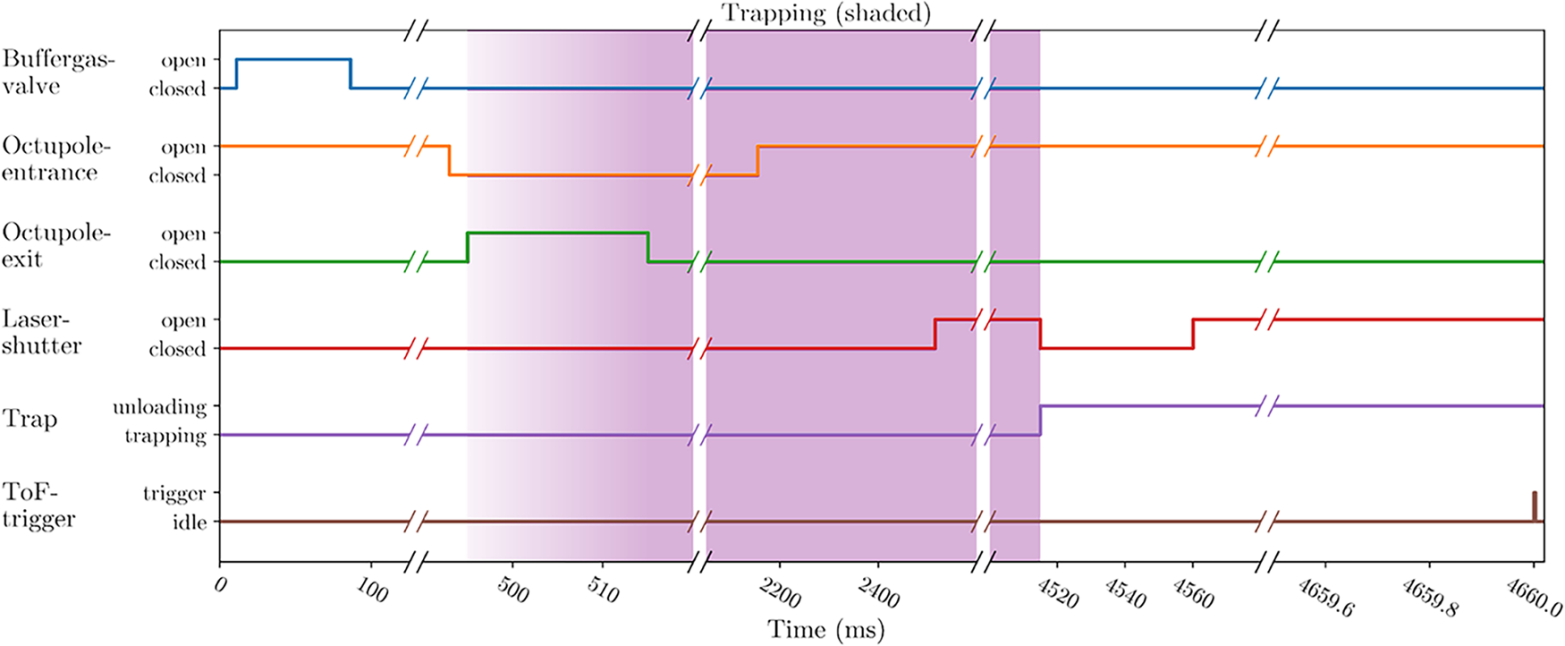
Exemplary set of timings of our experiment. Note that the *x*-axis of this plot is cut in multiple positions. Each cycle starts with a pulse of helium buffer gas being let into the trap. The ions, which were pretrapped in the octupole are then let into the main trap. These two timings are matched to each other so that the ions and the buffer gas arrive in the trap at the same time. Shortly before the exit of the octupole is opened, its entrance gets closed to prevent any nonthermalized ions from flying into the trap. When the entrance opens again, the pretrapping for the next experimental cycle begins. This usually overlaps with the current experimental cycle to speed up the experiment. Once the ions are trapped in the main trap, they thermalize via interaction with the buffer gas. After this, the laser shutter opens and the ions are irradiated for a selected time, unloaded from the trap, and guided toward the Wiley–McLaren time-of-flight mass spectrometer. It is triggered once the cold ions from the trap have reached it. Afterward the experiment restarts. The laser shutter reopens for a short time after the ions have left the trap to record the laser power.

To catch the ions in the cryogenic 16-pole wire trap, they are slowed-down and cooled by collisions with the cold helium buffer gas, which is pulsed into the trap. The timing of the buffer gas pulse is matched to the arrival of the ion bunch in the trap, which maximizes the number of trapped ions. Once the ions are trapped, the buffer gas diffuses out of the trap and is pumped away. This causes a lower buffer gas density while the ions are trapped, which increases the lifetime of ions inside the trap and reduces the collision-induced ion losses during its unloading. The temperature of the trap during all measurements was 3 K. After loading, the ions are stored in the trap for several seconds. Following a thermalization period of 2 s in the trap, the ions are irradiated with laser light for a set interaction time between 0.5 and 10 s that is chosen based on a maximum depletion of about 50%. In this way, the depletion signal was measured for laser wavelengths from 240 to 270 nm. The average laser power for every experimental cycle was measured using a reflection of the main beam off an uncoated fused silica window onto a calibrated photodiode (see Supporting Information for details).

Following the trapping, the ions are unloaded from the trap and guided into the linear Wiley–McLaren type time-of-flight mass spectrometer. To determine the relative depletion of the ion signal induced by the interaction with UV photons, the laser was blocked by a shutter for every other trapping and mass spectrum measurement cycle. The frequent remeasuring of the background reduces systematic errors that could otherwise be introduced due to intensity fluctuations of the nano-ESI source. We also recorded the wavelength dependent growth of the number of fragment ions, which are formed in the trap and also remain trapped within. For these measurements, the shutter was kept open for the entire measurement and every experimental cycle was recorded with UV photons inside the trap, since we did not observe any fragment ions without laser irradiation.

### Data Analysis

To obtain a quantity proportional to the ion number, the area under the observed [dAMP–H]^−^ peak in each mass spectrum is numerically integrated. For each cycle, where the UV light is present, a signal *i*_UVon_ is determined. The corresponding background signal is calculated from the mean of the mass spectra for cycles without laser light, which were recorded up to 60 s before or after the cycle that yielded *i*_UV on_. This time scale was found to be large enough to provide a suitable average, while it was smaller than the typical time scales of fluctuations of the nano-ESI source. For each signal *S*_*i*_UVon__ the relative depletion is then calculated as

1 $$S_{i_{\mathrm{rel}}}\left(\lambda ,t_{i}\right)=\frac{S_{i_{\mathrm{UVon}}}\left(\lambda ,t_{i}\right)}{S_{\mu_{i\mathrm{UVoff}}}\left(t_{i}\right)}=\frac{N}{N_{0}}=\exp\left(-k_{i}\left(\lambda \right)t_{i}\right)$$

where *N*_0_ is the initial number of [dAMP–H]^−^ ions and *N* is the number of [dAMP–H]^−^ ions remaining in the trap after exposure to UV light at a wavelength λ for time *t*_*i*_. *k*_*i*_(λ) is the [dAMP–H]^−^ decay constant at wavelength λ, based on a first-order decay process.

Measuments of *S*_*i*_rel__ are repeated ten times at each λ and fixed value of *t*_*i*_. Drifts in the laser power are compensated by normalizing against the mean laser power. Day-to-day variations in the overlap between the laser beam and ion cloud of up to 45% are compensated using repeated reference measurements taken at λ = 255 nm (*E* = 4.86 eV) on the same day (see Supporting Information for more details). This procedure yields the [dAMP–H]^−^ photofragmentation cross section relative to that at λ = 255 nm. Finally, a running mean of the scaled relative cross section weighted by the calculated error in each signal is calculated. These data are depicted in Figure 3.

**Figure 3:**
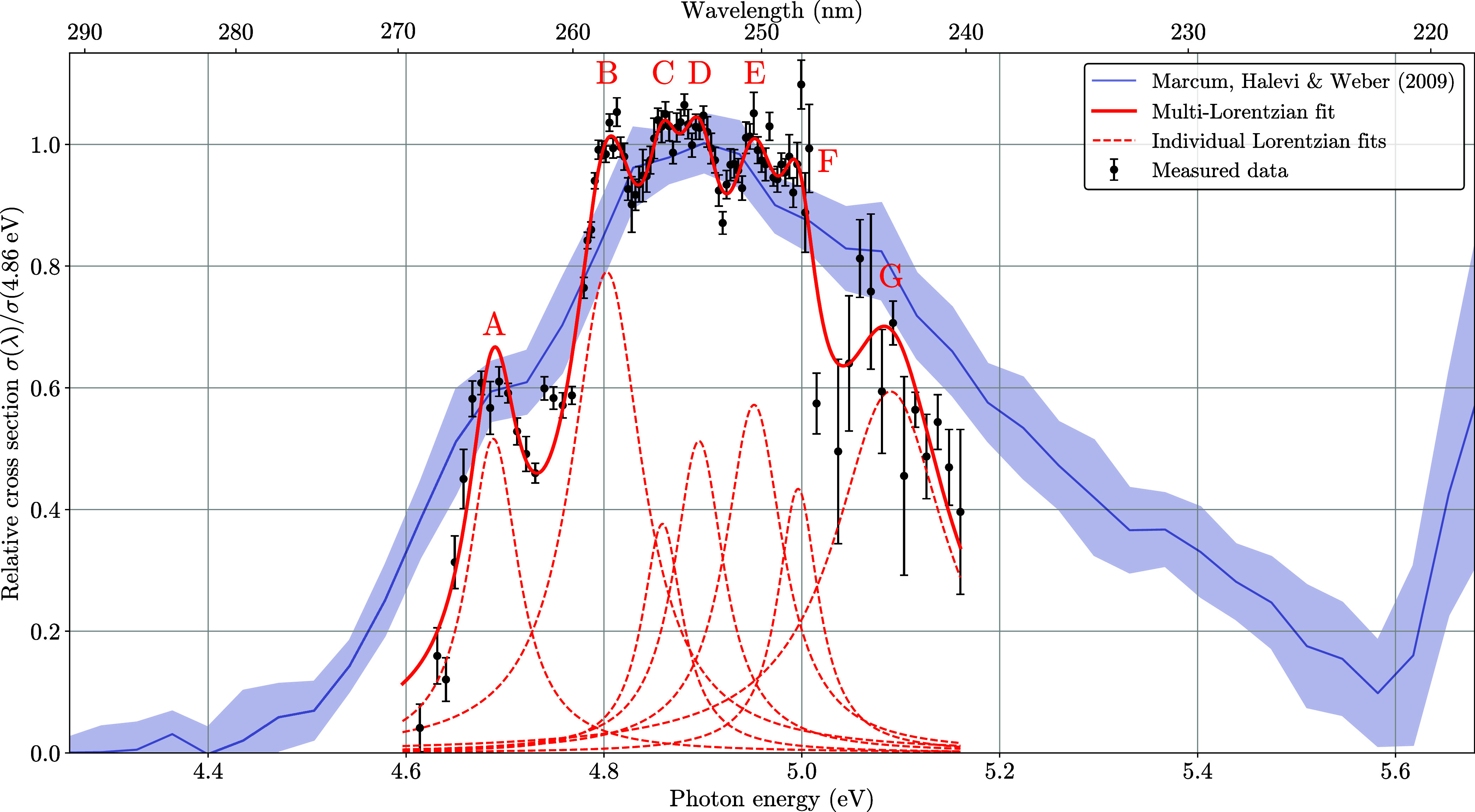
Measured relative photoabsorption cross section, depicted with 1 σ error bars. The red line displays a fit of seven Lorentzian peaks, one for each observed features A to G in the structure and a final one for the shoulder around 5.0 eV. Alongside our data we also plot the data recorded by Marcum, Halevi and Weber.^23^ We chose to plot this in a different representation than in their original paper, in order to present a less convoluted figure. For this purpose a running average was calculated over the data points of their work. The gray solid line shows the average of this data, the shaded area around it the 1 σ confidence interval.

Mass spectra of the [dAMP–H]^−^ photofragmentation products are recorded after exposing the ions in the trap to UV light for 0.5 to 2.5 s. This time is much shorter than the measured background lifetimes of the fragments in the trap, see Table S2. Therefore, the number of UV-induced ions of fragment F is modeled by a time-dependent growth

2 $$N_{F}\left(t,\lambda \right)=\epsilon_{F}\left(\lambda \right)\left(N_{0}-N\right)=\epsilon_{F}\left(\lambda \right)N_{0}\left(1-\exp\left(k\left(\lambda \right)t\right)\right)$$

where ϵ_F_(λ) is the branching ratio for fragment F at the wavelength λ. We have used the time-dependent decay of [dAMP–H]^−^ introduced in eq 1.

Accounting for the nonperfect and fragment-dependent detection efficiency γ_F_, the detected fragment F signal is written as

3 $$S_{F}\left(t,\lambda \right)=\gamma_{F}N_{F}\left(t,\lambda \right)=A_{F}\left(\lambda \right)\left(1-\exp\left(k\left(\lambda \right)t\right)\right)$$

where the pre-exponential terms are included into the fragment-specific factor *A*_F_(λ). The value of *A*_F_(λ) is determined from fitting *S*_F_(*t*, λ) at a constant wavelength with the value of *k*(λ) calculated from the measured [dAMP–H]^−^ relative cross section at λ and the [dAMP–H]^−^ cross section at 255 nm measured on the same day (see Supporting Information). In order to obtain a measure of ϵ_F_(λ) independent of *N*_0_ and γ_*F*_, the determined value of *A*_F_(λ) is divided by the mean value over all wavelengths.

### Theoretical Methods

The ground state structures of several conformers of the parent ion as well as the structures of the neutral and charged fragment molecules were optimized at the ωB97XD/aug-cc-pVDZ level of theory.^27^ The electronic excitation energies were then calculated for a set of representative parent ion conformers using time-dependent density functional theory, employing several functionals to approach the computational error, namely ωB97XD, CAM-B3LYP,^28^ BMK,^29^ and BHandHLYP, in combination with the aug-cc-pVDZ and aug-cc-pVTZ basis sets.

Due to the flexibility of the [dAMP–H]^−^ constituents, various conformers are possible, differing for example by the relative position of the phosphate group with respect to the adenosine as well as by the orientation of OH groups. We studied in total 14 such conformers, see Figure S2, six of which were taken as the lowest-energy ones identified in ref 14. All calculations were performed with Gaussian 16.^30^

## Results and Discussion

### Photofragment Cross Section Spectrum of [dAMP–H]^−^

The irradiation of the [dAMP–H]^−^ anions with UV light between 5.17 and 4.59 eV photon energy (240 and 270 nm), below its vertical electron detachment energy of 6.05(50) eV,^8^ leads to photofragmentation. The measured spectrum of the relative photoabsorption cross section is shown in Figure 3. Several distinct features can be resolved, which are labeled A to G.

To determine their location and width we created a fit of the sum of multiple Lorentzian peaks to the absorption spectrum. The equation used for the fit of a single Lorentzian was

4 $$y\left(x\right)=\frac{A\cdot \gamma^{2}}{{\left(x-x_{0}\right)}^{2}+\gamma^{2}}$$

and the sum of seven of these was created for the overall fit. All seven peaks are needed to create a matching fit to the data. However, due to the high error bars at the high-energy side of the spectrum, where the low laser intensity created larger fluctuations, we are not able to pinpoint the position of peak G very accurately. The obtained fit parameters are given in Table 1. The positions of the peaks do not change more than the fitting error if Gaussians are fitted instead of Lorentzians.

**Table 1:** Fit Parameters for the Fit Seen in Figure 3 ^a^

	*x*_0_ (eV)	γ (eV)	*A*
peak A	4.688(2)	0.031(5)	0.52(4)
peak B	4.803(3)	0.044(5)	0.79(6)
peak C	4.859(7)	0.025(15)	0.4(3)
peak D	4.896(7)	0.031(19)	0.5(3)
peak E	4.952(6)	0.04(2)	0.6(3)
peak F	4.996(6)	0.025(18)	0.4(3)
peak G	5.090(16)	0.07(3)	0.59(8)

a The equation used for the Lorentzian fits was eq 4.

The width of the features are not caused by the laser line width, which is less than 5 cm^–1^ (≈0.6 meV), or Doppler or pressure broadening, which are orders of magnitude smaller. In ref 31. the excited state lifetime of adenine is given as ≈40 fs, while that of 9-methyl adenine is ≈70 fs. The lifetime of [dAMP–H]^−^ has been measured by ref 15. They determined the decay to have two exponential components, where the faster one has a lifetime of < 60 fs. Transforming this to a lifetime broadening gives ≈20 meV. This lifetime thus explains the peak widths we observed. Because of the lifetime broadening, we chose Lorentzians to fit the individual peak shapes.

Comparing our spectrum to the one previously measured by Marcum, Halevi and Weber^23^ there is an overall excellent agreement, while several clear differences become evident. Their measurements shows one broad feature with “a slight shoulder” around 4.7 eV, but no substructure is observed. The entire peak in our spectrum is noticeably narrower than the one observed previously. The feature we label as Peak A, in Table 1 and Figure 3, coincides with the aforementioned “slight shoulder” from the previous measurement. The other peaks we observe fall into the broad main peak of that measurement. The employed laser systems have a comparable line width in both experiments. Therefore, we attribute the differences and the appearance of the additional features to the low temperature in our experiment, compared to the room temperature conditions in ref 23.

Using quantum chemical calculations, we identified the most stable structure (denoted A) to have a hydrogen bond between the OH group of the ribose part and an oxygen atom of the phosphate group. Conformers with a direct interaction between the phosphate group and the adenine moiety are by at least 0.54 eV higher in energy compared to the global minimum conformer, where the phosphate group interacts only with the ribose, see Figure S2. At the low temperature of the ion trap of 3 K, we only expect the lowest or the two lowest-energy conformers to be populated, as well as the electronic ground state and its lowest-lying vibrational levels.

We studied electronic excitations of [dAMP–H]^−^ using several methods of quantum chemistry and several conformers. With the energy of a single laser photon, excitation of [dAMP–H]^−^ is energetically possible via the very bright ππ* transition. Besides, two almost forbidden nπ* transitions are predicted by theory, see Figure S5. We assign the experimentally observed transition to the bright ππ* transition. Upon this excitation, the planarity of the adenine ring is broken, see Figure S6. Therefore, the excitation is likely to cause a vibrational excitation on top of the electronic excitation. A schematic of the ππ* transition together with the lowest-lying nπ* transition, calculated for conformer A, can be seen in Figure 4. The calculated energy for the ππ* transition is at 5.3 eV. The features we see in our spectrum are probably caused by excitations to different vibrational levels. Due to the size of [dAMP–H]^−^ the number of vibrational modes is very large. Therefore, it is likely that each of the features we observe and assign in Figure 3 is not a single line transition, but multiple transitions with similar energies. Given that the electronic excitation is located at the adenine moiety (see below) the Franck–Condon active vibrational modes are adenine vibrations. Our calculations hint in particular at the bending vibrations of the NH_2_ group.

**Figure 4:**
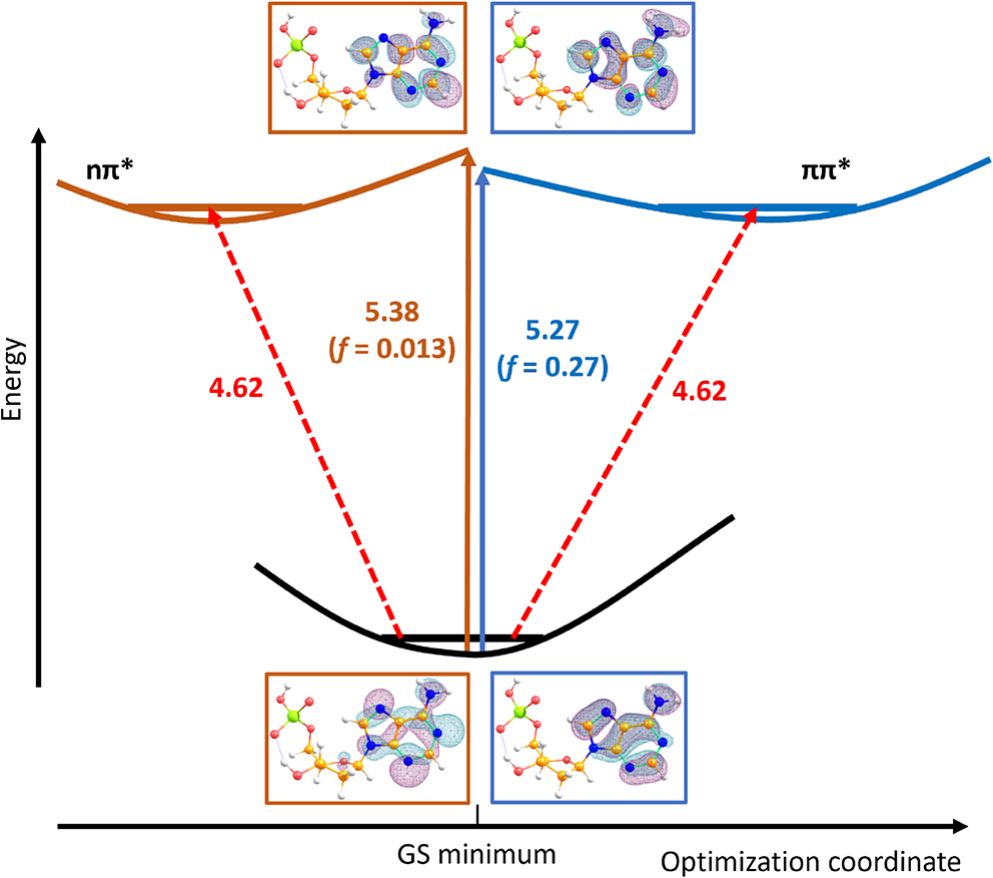
Scheme of [dAMP–H]^−^ photochemistry, showing the bright ππ* transition and the lowest-lying nπ* transition as calculated for conformer A. The transition energies are provided in eV as calculated at the ωB97XD/aug-cc-pVDZ level, *f* refers to the oscillator strength. For comparison, the analogous scheme for conformer G is shown in Figure S7.

Electronic spectra are predicted to be very similar, irrespective of the conformer, see Figure S4 and comparison between Figures 4 and S7 for more details. The reason is that the first bright transition of ππ* character takes place almost exclusively on the adenine moiety (see Figure 4), the influence of ribose and phosphate can be considered a perturbation. This transition is located at 5.2 to 5.3 eV in all found low-lying conformers (relative energies up to 0.8 eV), being either the lowest-lying transition or the second lowest one. The calculated value is overestimated by about 0.3 eV compared to the experimental position of the band maximum, and is consistent among all employed functionals, see Figure S4. For some functionals, in particular for CAM-B3LYP, the target state of this transition is mixed with a dipole-bound state, see Figure S5. We consider this to be a computational artifact due to the electron detachment energy lying close to the excitation energy, see Table S1 for an overview of electron detachment energies and excitations energies for the different methods used. No other considerably bright transitions are found in the region up to 6.0 eV (206 nm) where electron detachment already sets in. Among valence excitations, two *n*π* transitions are also notable, lying almost at the same energy as the ππ* transition, having however an approximately 10 to 20 times smaller intensity, see Figure S5. This picture is fully consistent with the one observed for neutral adenine.^18^

Our theoretical calculations show that, upon electronic excitation, there are several minima on the excited state potential energy surfaces to be reached. The adenine photochemistry is notoriously difficult to describe and advanced photochemical methods are needed.^17–22^ We located two minima that correspond to ππ* and twice nπ* in the photochemistry of the neutral adenine,^17,18^ see Figures 4 and S6. Following the ππ* state, the adenine molecule distorts as the NH_2_ group breaks the planarity of the molecule, with an excited state minimum lying about 0.6 eV below the vertical excitation energy. Following the *n*π* states, the adenine moiety stays almost planar, however with a slightly larger shift of about 0.8 eV along the optimization coordinate. The calculated energy difference between the ground state minimum and the ππ* excited state minimum of 4.6 eV matches well with the onset of the experimental spectrum.

### Fragment Analysis

To investigate whether the peaks we observed favor one of the fragmentation paths, we performed measurements of the growth of several fragments at different photon energies. These were the five fragments that were previously observed under photofragmentation of [dAMP–H]^−^:^11^ PO_3_^–^ (79 Da), H_2_PO_4_^–^ (97 Da), [A–H]^−^ (134 Da), [dAMP–H–A–H_2_O]^−^ (177 Da) and [dAMP–H–A]^−^ (195 Da), with A symbolising the adenine molecule. An example mass spectrum of these fragments, as well as their structures are shown in Figure 5. The result of this analysis is presented in Figure 6. The depicted yields for each fragment are normalized to the average signal of this specific fragment over the entire wavelength range. Therefore, the intensities of the fragments cannot be directly compared with each other, due to the different coupling efficiencies of each mass into the mass spectrometer, which are difficult to quantify. Dissociation energies for all observed channels are calculated to lie below 2.1 eV (see Figure S3), making all of them accessible upon absorption of a single photon.

**Figure 5:**
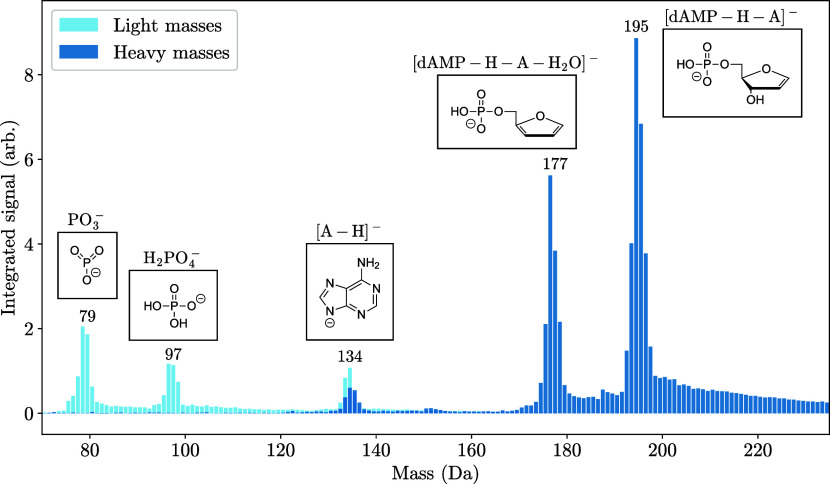
Combined mass spectrum of the detected [dAMP–H]^−^ fragments. This is taken using two different time-of-flight measurements between the trap and Wiley–McLaren plates (light and dark blue) since we are unable to couple all ion masses simultaneously into the mass spectrometer (refer to Supporting Information for details). Differences in the coupling efficiencies for the masses prevent the peak intensities from being directly comparable to each other.

**Figure 6:**
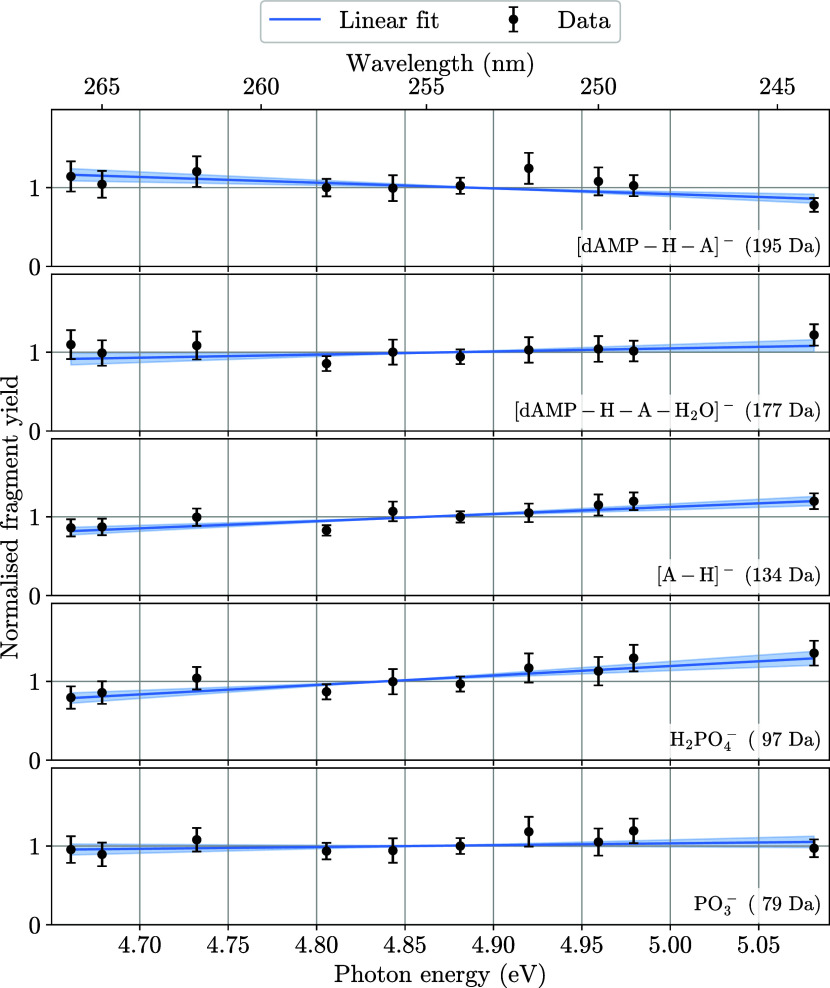
Wavelength-dependent fragment yields for the five fragments that were observed for the photofragmentation of [dAMP–H]^−^. The yield of each fragment has been normalized to its own average over all wavelengths, to show wavelength dependent changes of each fragment. We additionally include linear fits of the fragment data with the shaded region depicting the fit variation as we vary the fit parameters by up to one σ of their error.

The relative yields in Figure 6 are fit to linear functions to allow for a quantitative assessment of their dependence on photon energy. From these fits one can see that the yield of the heaviest fragment [dAMP–H–A]^−^ (195 Da) decreases with photon energy, while the fragments H_2_PO_4_^–^ (97 Da) and [A–H]^−^ (134 Da) show a clear increase. The other two fragments, PO_3_^–^ (79 Da) and [dAMP–H–A–H_2_O]^−^ (177 Da), show no significant dependence on photon energy. The data suggest that the fragmentation pathway of the heaviest fragment has the lowest threshold or intermediate energy barrier. In this case its production becomes less likely at higher photon energies, where other pathways with higher appearance energies open up.

References 5 and 7 performed CID experiments and for both experiments the most abundant fragment of [dAMP–H]^−^ was [dAMP–H–A]^−^. The lists of fragments they observed were the same as in our work. As stated in ref 11, the photodissociation happens on the electronical ground state, as it does in CID. In that study they performed a photofragmentation study at 4.71 eV photon energy and also observed [dAMP–H–A]^−^ as the most abundant fragment.^11^ A further photofragmentation study at 4.77 eV photon energy by ref 23 also observed [dAMP–H–A]^−^ as the most abundant fragment. These two studies also observed the other fragments that we observed in the present work. Reference 11 showed that [dAMP–H–A]^−^ and [A–H]^−^ are formed by the same two body breakup, where the negative charge can remain on either of the two fragments. This could explain why the two fragment’s formation rates (134 and 195 Da) are anticorrelated, with respect to the photon energy. PO_3_^–^ is also formed in a two body breakup with the other part of this fragmentation being neutral and therefore undetectable with our setup. Reference 11 also concluded that the fragments H_2_PO_4_^–^ and [dAMP–H–A–H_2_O]^−^ are each created in sequential breakups. H_2_PO_4_^–^ is formed in the first step of a two step process, while the neutral cofragment dissociates further. [dAMP–H–A–H_2_O]^−^ is formed when [dAMP–H]^−^ looses a water molecule, before the remaining molecule breaks into [dAMP–H–A–H_2_O]^−^ and a neutral A molecule. Our calculations additionally suggest a competing pathway of formation of [dAMP–H–A–H_2_O]^−^, where the adenine molecule is lost first, and a water molecule evaporates in the second step (see Figure S3), in contrast to this pathway being excluded in ref 11.

In our analysis, it was found that two fragments, [A–H]^−^ and [dAMP–H–A–H_2_O]^−^, are photoactive (see Figures S8 and S9 and Table S2). This can be expected for [A–H]^−^ since the adenine base is known to absorb UV photons.^31,32^ Furthermore, the UV action spectra of the protonated adenine cation (AH^+^) and the protonated 2’-adenosine 5′-monophosphate cation (dAMPH^+^) have been shown to be “almost identical”,^13^ which supports the claim that the adenine part is responsible for the photoabsorption. The second fragment, [dAMP–H–A–H_2_O]^−^, contains a furan-like ring with an additional double bond that is not present in [dAMP–H]^−^, but is created during fragmentation, as also suggested in ref 7. This is expected to reduce the energy required for photoexciting this fragment, which explains its photoactivity in the studied wavelength range.

### Absolute Photodetachment Cross Section

For this measurement we measured the UV lifetime of [dAMP–H]^−^ and I^–^ under equal experimental conditions. This allows us to scale the relative photofragmentation cross section of [dAMP–H]^−^ to the known absolute photodetachment cross section of I^–^. Based on this comparison the absolute photofragmentation cross section of [dAMP–H]^−^ at 255 nm (4.86 eV) is 1.0(4) × 10^–16^ cm^2^. The value is approximately three times larger than the photodetachment cross section of I^–^, which is taken to be 3.5(6) × 10^–17^ cm^2^ from a combination of the values of refs 33 and 34. The stated error bar is mostly caused by the systematic error given by a slowly time-varying overlap between the UV laser beam and the trapped ion cloud. Our measured value is in agreement with the absorbance of dAMP in solution, which was measured to be 15 l/mmol/cm or about 5.8 × 10^–17^ cm^2^.^35^

## Conclusion

In the present study, the UV-induced photofragmentation of [dAMP–H]^−^ was investigated inside a cryogenic radiofrequency ion trap. By isolating the nucleotide, we studied its inherent photophysical behavior without interference from other molecular factors, which provides a crucial reference point for understanding how these factors alter the photodissociation behavior in vivo. The relative photofragment cross section was measured between 5.17 and 4.59 eV photon energy (240 and 270 nm). The spectrum confirms the previous results of ref 23., but provides a significantly higher resolution and therefore shows several new features. We attribute the overall narrower spectrum and the resolved features to the low internal temperature of the ions in the cryogenic trap. Specifically, below 10 K only one or at most two conformers of very similar structure are expected, based on the relative energies of the lowest-energy conformers. The seven different features resolved in the spectrum are fitted with a sum of Lorentzians. The widths of these peaks are close to the expected width based on the lifetimes of the excited states of adenine and 9-methyl adenine.^31^ Our theoretical calculations predict a bright ππ* transition located at the adenine moiety in the energy range where the photodissociation spectrum was measured, but an assignment of the exact vibronic states for the features we observed was not possible.

The analysis of the five detected photofragments shows that there is no strong preference for a single fragment at any of the studied wavelengths. We do, however, observe slightly different trends of the fragment yield as a function of photon energy. H_2_PO_4_^–^ and [A–H]^−^ show the strongest tendencies to increase with higher photon energy, while the tendency is reversed for [dAMP–H–A–H_2_O]^−^. Two of the fragments are themselves photoactive and decay into secondary products. This is expected for the first one, [A–H]^−^, which is the photoactive part of [dAMP–H]^−^. For the second photoactive fragment, [dAMP–H–A–H_2_O]^−^, we attribute the photoactivity to a rearrangement during the fragmentation, which creates a ring structure with a double bond as the photoactive part.

Finally, we also determined the absolute photofragmentation cross section for [dAMP–H]^−^ photofragmentation by comparing its measured relative cross section to the relative cross section for photodetachment of I^–^ in the same trap. A value of 1.0(4) × 10^–16^ cm^2^ was obtained, about three times larger than the I^–^ photodetachment cross section, and in agreement with the absorption coefficient of dAMP in solution.

## Supplementary Material



## Notes

Detailed description of the experimental setup, the data analysis methods, and additional experimental and theory data (PDF)

## §

Department of Chemical Engineering, KTH Royal Institute of Technology, 10044 Stockholm, Sweden
