# Supplementary material for: Ultraviolet Photodissociation Spectroscopy of [dAMP–H]− at Low Temperature
Source: J Phys Chem A. 2025 Dec 2;129(50):11571–9. doi: 10.1021/acs.jpca.5c06525 (PMC12720237; doi:10.1021/acs.jpca.5c06525)
Supplement: Supplementary file 1 [file jp5c06525_si_001.pdf]

Supplementary Information – Ultraviolet Photodissociation Spectroscopy of $[\mathrm{dAMP-H}]^-$ at Low Temperature

Christian Sprenger,[†] Samuel J. M. White,[†] Miriam Westermeier,[†] Gabriel Schöpfer,[†] Franziska Dahlmann,[†,¶] Uma Namangalam,[‡] Salvi Mohandas,[‡] Sunil Kumar S.,[‡] Eric S. Endres,[†] Milan Ončák,[†] and Roland Wester[*,†]

†*Institut für Ionenphysik und Angewandte Physik, Universität Innsbruck, Technikerstraße 25, 6020 Innsbruck, Austria*

‡*Indian Institute of Science and Technology Tirupati, Department of Physics, Srinivasapuram, Yerpedu Mandal, Tirupati Dist, Andhra Pradesh, India – 517619*

¶*Current address: Department of Chemical Engineering, KTH Royal Institute of Technology, 100 44 Stockholm, Sweden*

E-mail: roland.wester@uibk.ac.at

1 Experimental details

1.1 ESI setup

The needles for the nano-ESI are pulled from borosilicate capillaries with a length of 100 mm, 1 mm outer diameter and 0.78 mm inner diameter (Harvard Apparatus 30-0035) using a Sutter Instrument P-1000 micropipette puller. During this process we create two needles of approximately equal length with closed ends, which we break open using tweezers under a microscope.

The 70 mm-long transfer capillary is made from 316 stainless steel with 1/16 inch outer diameter 500 µm inner diameter (Vici T20C20D). The capillary leads into the first source chamber where the differential pumping gives a pressure between 10 mbar and 25 mbar. We tune this pressure by adjusting a valve fitted before an Edwards XDS35 pump and empirically find that the pressure must remain within this range for ions to be efficiently transferred through the source. Beam Dynamics skimmers are used to separate the two source chambers and the second source chamber from the octupole chamber which allow further differential pumping. An Edwards XDS100B backed by an Edwards nXDS15i is used to pump this second chamber resulting in a pressure of 0.5 mbar. The ion transport is found to be insensitive to the pressure in this chamber, however it has a significant influence on the pressures that can be achieved in the subsequent chambers.

Tubular lenses are located behind each skimmer for tuning the ion beam. They have inner diameters of 5 mm (5.5 mm) and lengths of 3 mm (2.75 mm) for the first (second) lenses, respectively. The tube of the second lens protrudes into the octupole ion guide and serves as its entrance lens. The pressure in the octupole chamber is $\approx 5 \times 10^{-4}$ mbar. The octupole ion guide is custom-built with a rod diameter of 3 mm and inscribed radius of 4.03 mm. At the end of the ion guide is a 3 mm-diameter, 8 mm-long tube lens known as the "conductance limiter". It serves as another differential pumping stage as well as an endcap to enable pre-trapping in the octupole while also being used to focus exiting ions into the

quadrupole. The quadrupole is also custom-built with rods consisting of straight sections of gas line with an outer diameter of 6 mm arranged with an inscribed radius of 2.6 mm. The quadrupole can be used as an ion guide, or a low resolution mass filter.

After the quadrupole the ions pass a segmented lens where the voltage of each of the four segments can be controlled individually to simultaneously focus and deflect the ions into the trap. The vertical position of the trap changes by several hundred micrometers between operation at room temperature and at 3 K due to thermal expansion, such that this ability to deflect the ions is crucial for permitting operation in both regimes. The remaining setup is the same as that described in Ref.[1]

1.2 Optical setup

A diagram of the optical setup used to manipulate and monitor the radiation emitted by the Ekspla NT 242-SH/SF laser system is shown in Figure S1. A Galilean telescope (Thorlabs LA4924-UV, $f = 175$ mm and Thorlabs LC4252-UV, $f = -30$ mm lenses) was used to reduce the size of the elliptical beam to a width of ≈ 3 mm in the smaller dimension before focussing with a third lens (Thorlabs LA4579-UV, $f = 300$ mm) into the chamber.

Broadband (245 nm to 390 nm) dielectric coated mirrors (CVI Laser Optics MAXBRIte, MPQ-245-390-2506M) were used for all mirrors except the third mirror where a 266 nm laser line mirror (CVI Laser Optics Y4-1025-45) was used. This enabled the removal of 532 nm light output from the laser.

The reflection from a fused silica mirror blank (CVI Laser Optics PM-1025-UV) onto a photodiode detector (Coherent 1098313) is used in order to monitor the laser power. The resultant photodiode voltage is read out from a power meter (Coherent FieldMate 1098297) and calibrated against a thermal power sensor (Thorlabs S401C) placed immediately before the chamber at wavelengths from 230 nm to 290 nm. The power calibration was repeated > 10 times over different days in order to collect calibration data for a range of different laser powers at each wavelength. We further describe how these data are used in section 3.1.

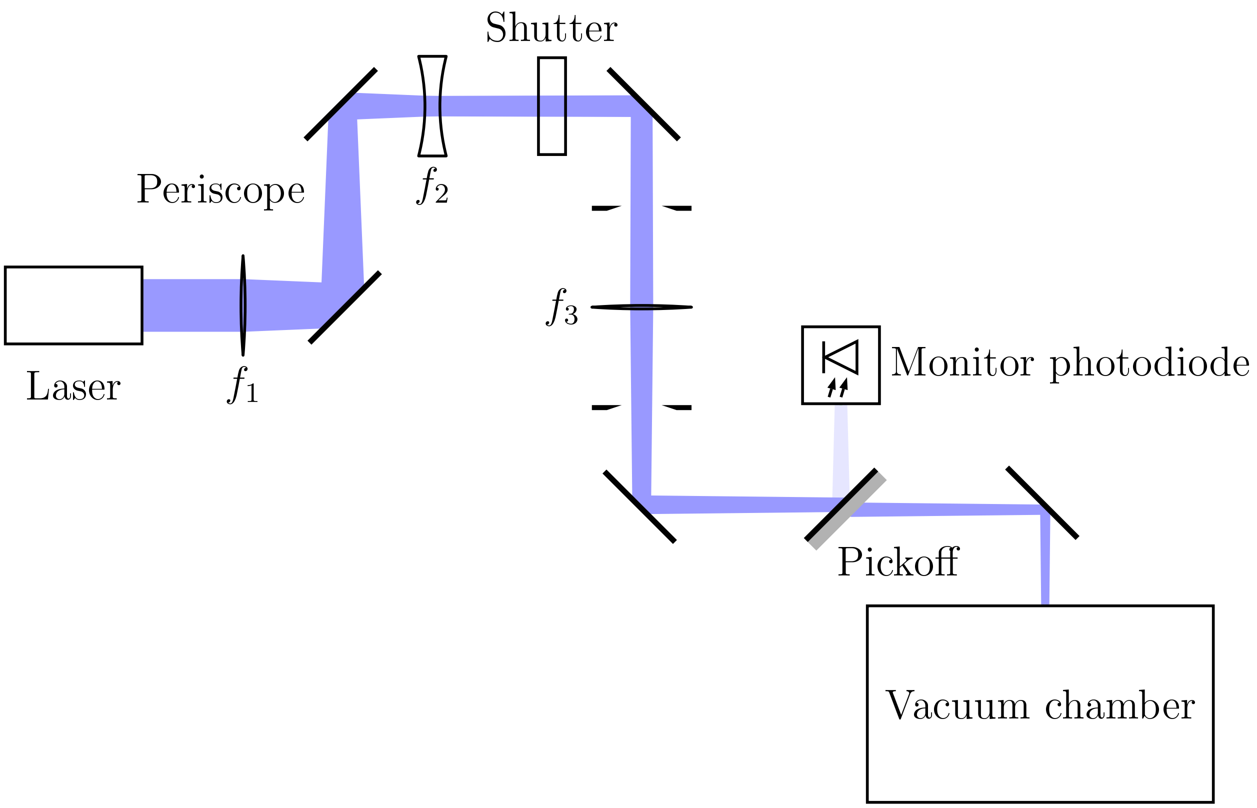

Figure S1: Diagram of optical setup. The lenses in the setup are used to increase the beam intensity in the chamber. We chose $f_1 = 175\,\text{mm}$, $f_2 = -30\,\text{mm}$ and $f_3 = 300\,\text{mm}$ with the first two lenses placed to form a Galilean telescope.

The final mirror before the vacuum chamber was placed on a translation stage allowing horizontal motion parallel to the trap axis at a fixed height. The mirror position was initially optimised on the overlap between the laser and ion cloud and then kept fixed for all the measurements taken.

1.3 Wiley-McLaren delay times

After unloading the trap, the ions travel over a distance of $\approx 28\,\text{cm}$ towards the Wiley-McLaren plates. During this time-of-flight the ion cloud acquires a longitudinal mass-dependent time distribution and extends in size such that we are unable to transfer all of the ions with the Wiley-McLaren plates into the time-of-flight tube.

As such we need to select an appropriate delay time between the trap unloading and

pulsing of the Wiley-McLaren plates dependent on the mass of the ions to be analysed in the mass spectrum. For the lighter ions (${PO_3}^-$ and ${H_2PO_4}^-$) this is chosen to be between 89 µs to 91 µs and for the heavier ions ($[\mathrm{dAMP{-}H{-}A{-}H_2O}]^-$ and $[\mathrm{dAMP{-}H{-}A}]^-$) we choose between 107 µs to 108 µs. Signal from the $[\mathrm{A{-}H}]^-$ fragment is visible for both of these delay choices as shown in Figure 5.

2 Theory

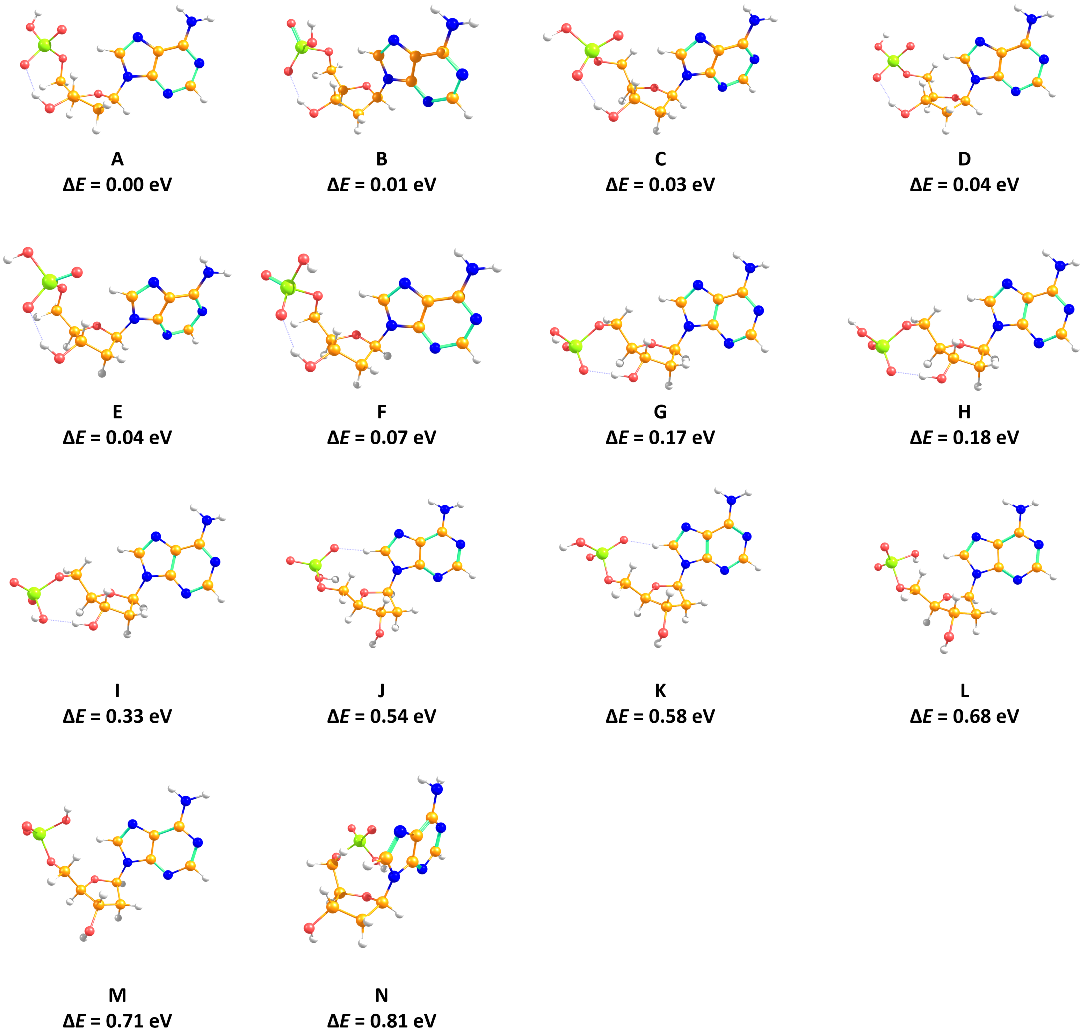

Figure S2: Located conformers of $[\text{dAMP}-\text{H}]^-$ as optimized at the ωB97XD/aug-cc-pVDZ level, along with their relative energies.

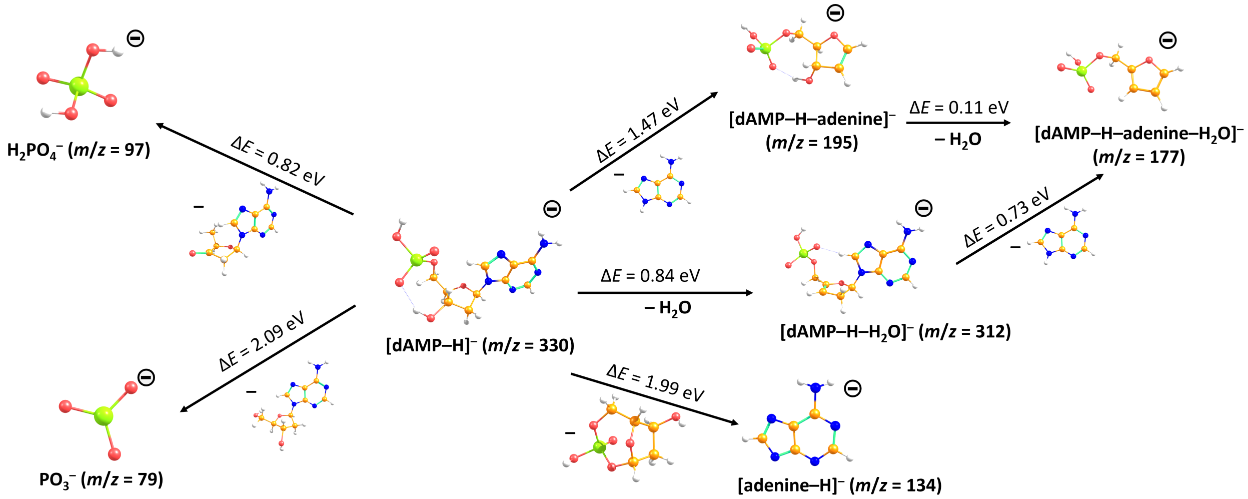

Figure S3: Calculated fragmentation channels for conformer A along with reaction energies as obtained at the ωB97XD/aug-cc-pVDZ level.

Table S1: Vertical excitation energy of the bright $\pi\pi^\star$ transition and vertical detachment energy in conformer A. Structure optimized at the ωB97XD/aug-cc-pVDZ level of theory.

Method	Energy of the $\pi\pi^\star$ transition (eV)	Vertical detachment energy (eV)
ωB97XD/aug-cc-pVDZ	5.27	5.68
ωB97XD/aug-cc-pVTZ	5.25	5.66
CAM-B3LYP/aug-cc-pVDZ	5.29	5.71
CAM-B3LYP/aug-cc-pVTZ	5.27	5.73
BHandHLYP/aug-cc-pVDZ	5.40	5.56
BMK/aug-cc-pVDZ	5.31	5.58

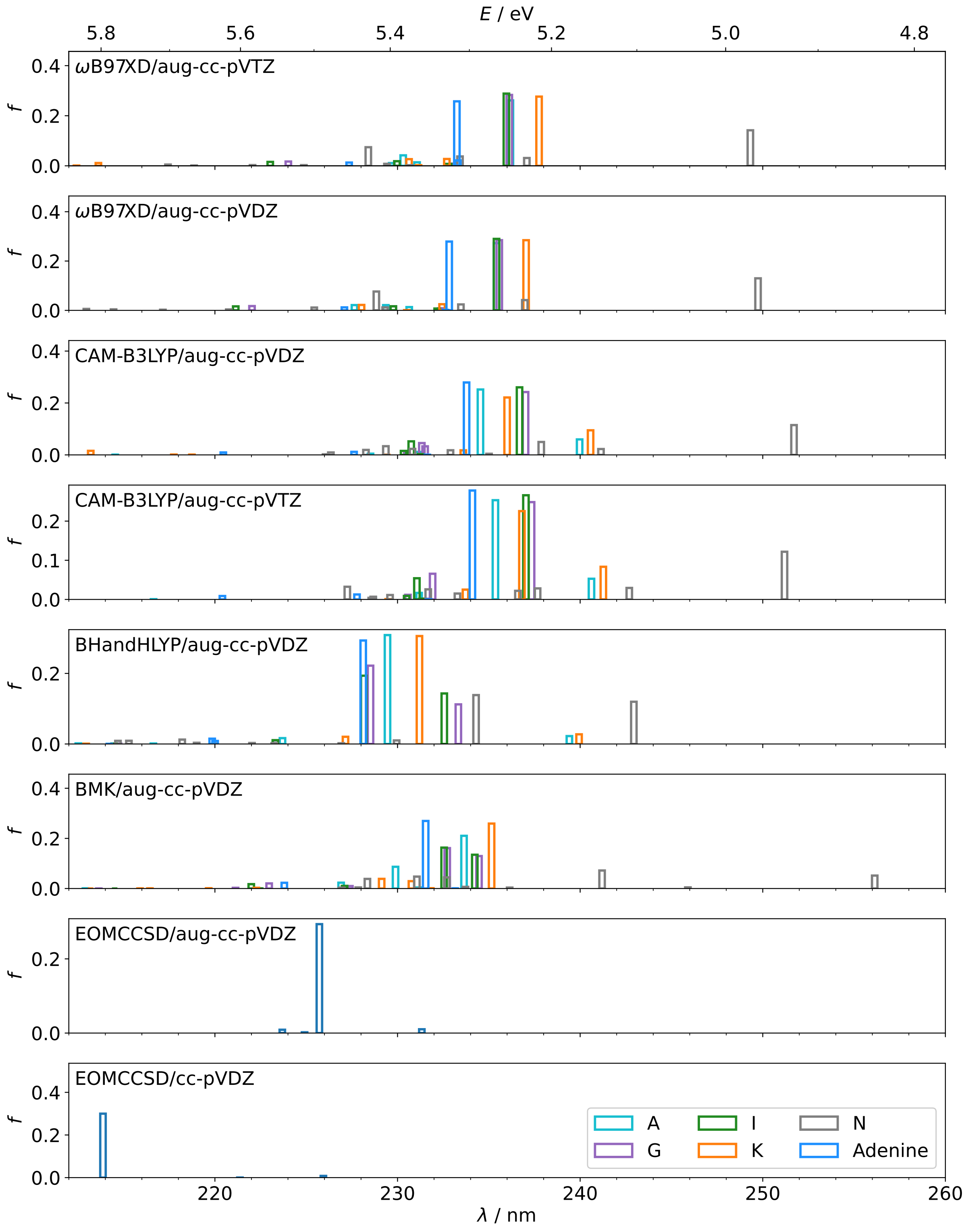

Figure S4: Comparison of electronic transitions calculated for five representative conformers of $[\text{dAMP}-\text{H}]^-$ and the neutral adenine molecule at various levels of theory in structures optimized at the ωB97XD/aug-cc-pVDZ level. The first ten excited states were calculated apart from EOMCCSD/aug-cc-pVDZ, for which four were considered.

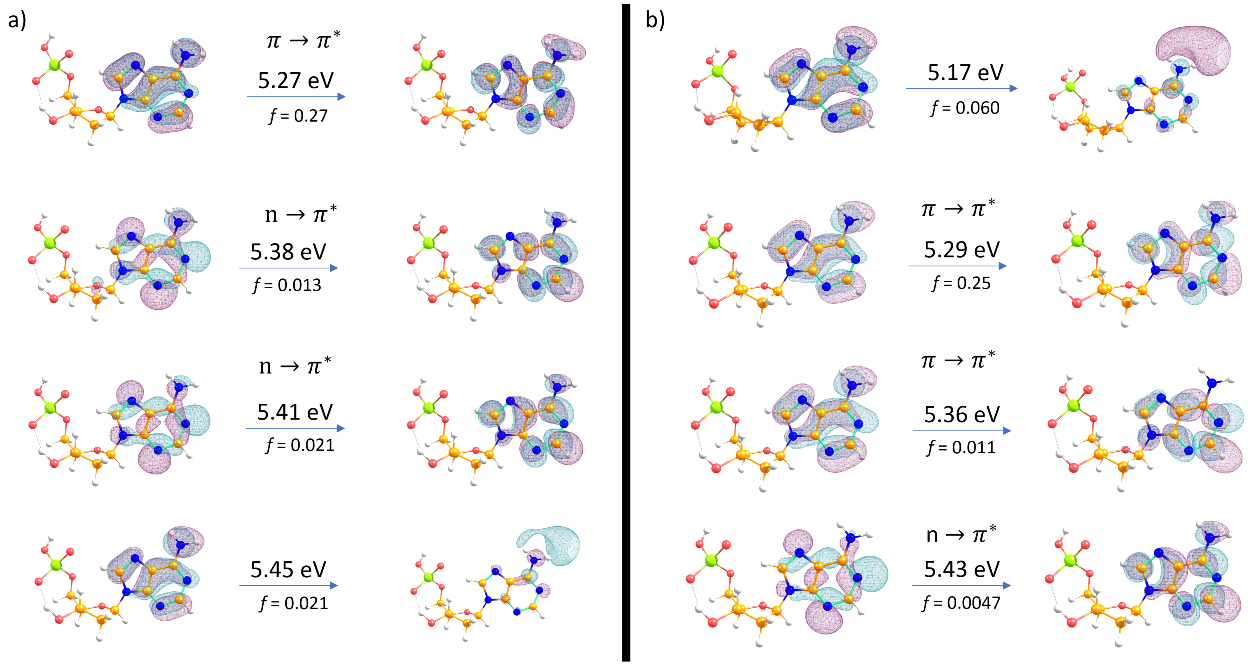

Figure S5: First four vertical transitions in the conformer A minimum structure. Shown are the orbitals associated with the transitions, along with their characters, the vertical excitation energies and oscillator strengths as obtained at the a) ωB97XD/aug-cc-pVDZ and b) CAM-B3LYP/aug-cc-pVDZ level. The first two transitions in a) correspond to the ππ* and nπ* transitions discussed in the main text. The relatively high intensity for the first transition in b) is supposedly a computational artifact due to mixing of a transition into the dipole-bound state with the high-intensity $\pi\pi*$ transition.

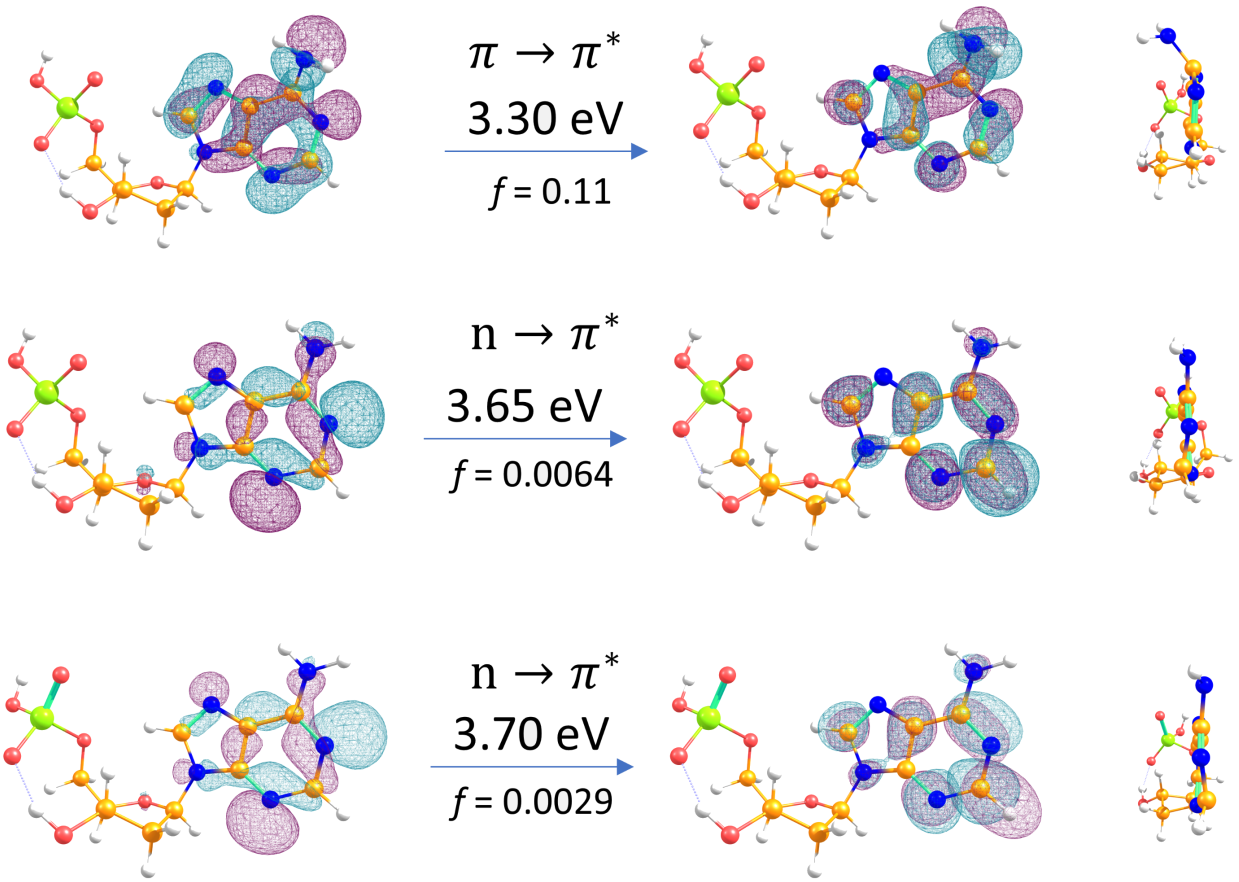

Figure S6: First three vertical transitions in conformer A, after optimization in the respective excited state. Shown are the orbitals associated with the transitions, along with their characters, the vertical excitation energies and oscillator strengths as obtained at the ωB97XD/aug-cc-pVDZ level. The first two transitions correspond to the $\pi\pi^*$ and $n\pi^*$ transitions discussed in the main text. From comparison with Figure S5 it can be seen that the character does not change upon optimization in the respectives excited states. The side views of the structures are also given to show the breaking of the planarity of the adenine moiety upon $\pi\pi^*$ transition.

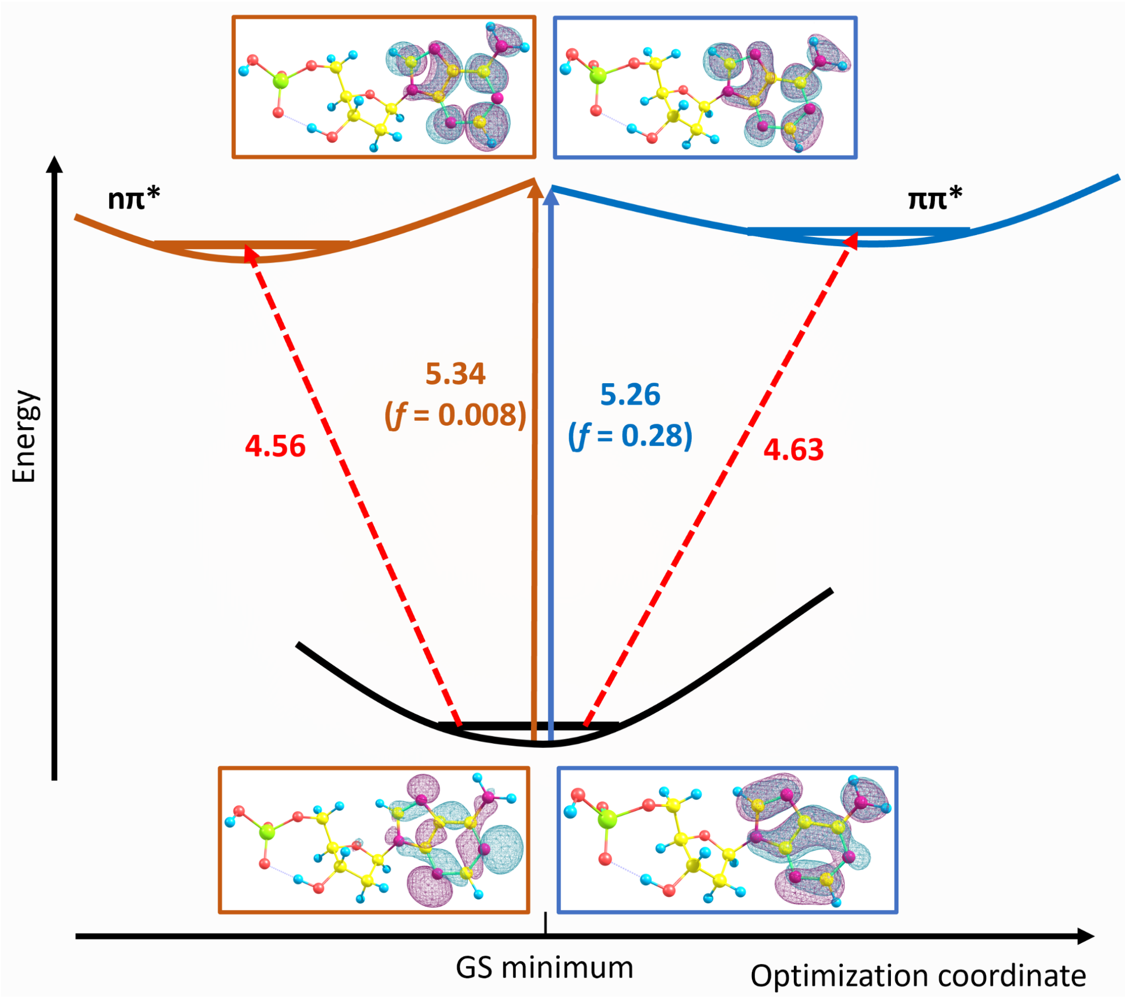

Figure S7: Scheme of [dAMP−H]$^-$ photochemistry, showing the bright ππ* transition and the lowest-lying nπ* transition as calculated for conformer G. The relative energies are provided in eV as calculated at the ωB97XD/aug-cc-pVDZ level.

3 Data analysis

3.1 Laser power normalisation

We record the voltage from the monitoring photodiode during each measurement and use the difference between the voltages in the measurements with and without the laser light to remove the effect of background light. For technical reasons, this measurement is taken at the end of the experimental cycle when we record the mass spectrum, hence why we open the laser shutter again after unloading the ions as shown in Figure 2. The relation between this voltage difference and the laser power was found to be strongly wavelength-dependent due to a strong wavelength-dependence of the optics used.

To account for this dependence, we measured the photodiode voltage difference and laser power before the chamber at 0.2 nm wavelength intervals at a range of different laser powers taken over multiple days. A linear fit of these data was then performed at each calibration wavelength before computing an weighted, smoothing interpolation of the two fit parameters over the measured wavelengths using a spline. The weights for this smoothing interpolation were computed using the errors of the fit parameters at each wavelength. This procedure reduces the noise in the calibration and allows for reliable extraction of the laser power for data taken at wavelengths between those for which calibration data was taken. We disregard the unknown losses due to back reflections and non-perfect transmission through the chamber viewport since we expect these fractional losses to be constant and will thus cancel in our relative measurements.

The laser power P for each measurement at a wavelength λ is converted into a photon flux $\Phi(P, \lambda) = P\lambda/(hc)$ for h the Planck constant and c the speed of light. We take the Ansatz that for a measurement i the $[\mathrm{dAMP-H}]^-$ decay rate takes the form $k_i(\lambda) = \Phi(P, \lambda)\alpha\sigma(\lambda)$ for α a geometric overlap factor between the laser beam and the trapped ions and $\sigma(\lambda)$ the absolute photofragmentation cross section. For each measurement $[\mathrm{dAMP-H}]^-$ ion signal

measurement, we compute the value of

$$S_i = \frac{-\log\left(S_{i_{\mathrm{rel}}}\right)}{\Phi(P,\lambda)t_i} = \frac{k_i(\lambda)t_i}{\Phi(P,\lambda)t_i} = \alpha\sigma(\lambda), \tag{S1}$$

where $S_{i_{\mathrm{rel}}}$ is as defined in equation (1) of the main article and t_i is the time the UV light is on during the measurement. This thus removes the dependence on the laser power from the measurement.

3.2 Daily $[\mathrm{dAMP-H}]^-$ decay reference

Each day we record the decay of $[\mathrm{dAMP-H}]^-$ ions under UV light at 255 nm for at least five different times and power-normalise the resultant signal by taking

$$S_{\mathrm{ref}} = -\log\left(S_{i_{\mathrm{rel}}}\right)/\Phi(P, 255\,\mathrm{nm}) = \alpha\sigma_{\mathrm{ref}}\,t_i, \tag{S2}$$

for the quantities as defined for equation (S1) and with $\sigma_{\mathrm{ref}} := \sigma(255\,\mathrm{nm})$. The gradient of a linear fit completed against these data allows us to extract $\alpha\sigma_{\mathrm{ref}}$. The relative cross section σ_{rel}, independent of α, is found by taking

$$\sigma_{\mathrm{rel}} = \frac{S_i}{\alpha\sigma_{\mathrm{ref}}} = \frac{\sigma(\lambda)}{\sigma_{\mathrm{ref}}}, \tag{S3}$$

for S_i as defined in equation (S1).

The value of α may change on a day-to-day basis due to changes in the static charge distribution around the trapping region resultant from neutral gasses freezing in different places in the cooled trap and subsequently accumulating charges. Additional changes in this charge distribution throughout the day are also observed and corrected for using an empirically determined scaling factor between 0.85 and 1.426 (mean value 1.13, standard deviation 0.2).

3.3 Fragment lifetimes

We create fragments in the trap by exposing $[dAMP-H]^-$ in the trap to UV light at 255 nm for either 3.0 s (when studying $[dAMP-H-A]^-$, $[dAMP-H-A-H_2O]^-$ and $[A-H]^-$) or 10.0 s (for $H_2PO_4{}^-$ and $PO_3{}^-$). The longer time is used for the lighter fragments to increase the weak ion signal we see for these. We then vary the time which we subsequently hold the fragments in the trap in order to measure their "natural" lifetimes.

As shown in Table S2 and Figure S8, all fragments have lifetimes in the trap of longer than 10 s. This was determined by completing exponential fits on the ion signal in the time period after we turned the UV light off. We also see that the lighter fragments are lost much faster than the heavier ones, which we expect as we are operating the trap with an RF frequency optimised for the best possible trapping of $[dAMP-H]^-$.

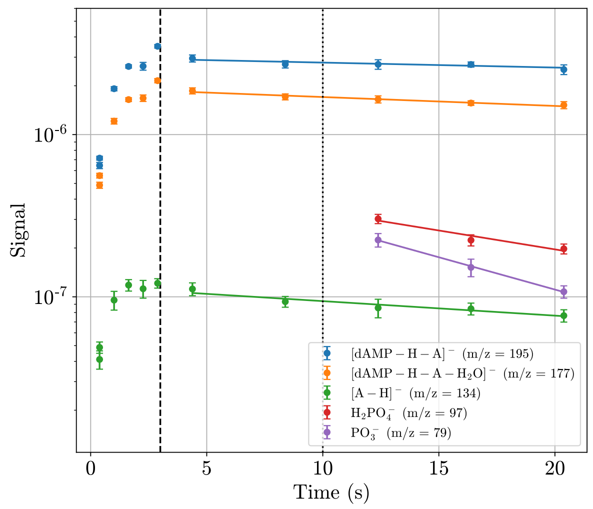

Figure S8: Fragment natural lifetimes in the trap. UV light at 255 nm is on for up to 10.0 s (dotted line, $H_2PO_4{}^-$ and $PO_3{}^-$ only) or 3.0 s (dashed line, all other fragments) or it is turned off.

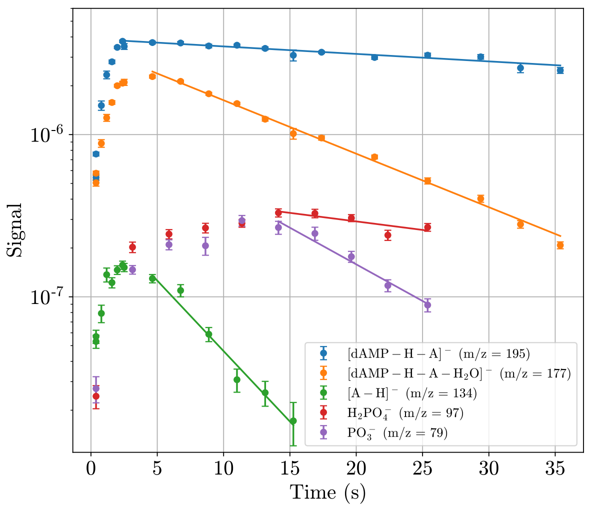

Figure S9: Fragment signal development and lifetimes under continuous exposure to UV light at 255 nm

We then repeat this procedure keeping the UV light always on. We see in Table S2 and Figure S9 that the lifetimes for the $[dAMP-H-A-H_2O]^-$ and $[A-H]^-$ fragments are

drastically reduced to 13.2(2) s and 5.0(4) s respectively and that for $H_2PO_4^-$ increases from 19(4) s to 41(11) s while those for the other fragments remain unaffected. We expect that the loss in $[A-H]^-$ observed during the UV-light exposure could be the result of photodetachment since the energy of the used 255 nm-light (4.86 eV) is above the predicted deprotonated adenine electron affinity of 3.50 eV.[2]

The increased lifetime of $H_2PO_4^-$ with longer UV light exposure might suggest that the loss of the $[dAMP-H-A-H_2O]^-$ fragment involves further fragmentation with $H_2PO_4^-$ as one of the fragmentation products. It is however difficult to draw robust conclusions given the limited number of data points collected for the $H_2PO_4^-$ natural lifetime and the consequent large uncertainty in the fitted exponential decay curve.

Table S2: Fitted lifetimes of $[dAMP-H]^-$ fragments from data shown in Figures S8 and S9.

Fragment	m/z	Lifetime without UV light (s)	Lifetime under UV light (s)
$[dAMP-H-A]^-$	195	132(85)	94(7)
$[dAMP-H-A-H_2O]^-$	177	79(22)	13.2(2)
$[A-H]^-$	134	49(17)	5.0(4)
$H_2PO_4^-$	97	19(4)	41(11)
PO_4^-	79	11(2)	9.6(9)

3.4 Fragment signal normalisation

In order to remove the initial $[dAMP-H]^-$ ion number N_0 and fragment detection efficiency γ_F within $A_F(\lambda)$ shown in equation (3) of the main article, we divide by the mean value of $A_F(\lambda)$ over all measured wavelengths for fragment F. This implicitly assumes that N_0 remains constant during each set of measurements taken. From the data taken for the $[dAMP-H]^-$ spectrum measurements, we estimate the standard deviation in N_0 to be 12(1) % and see no significant effect of measurement duration on this value as shown in Figure S10.

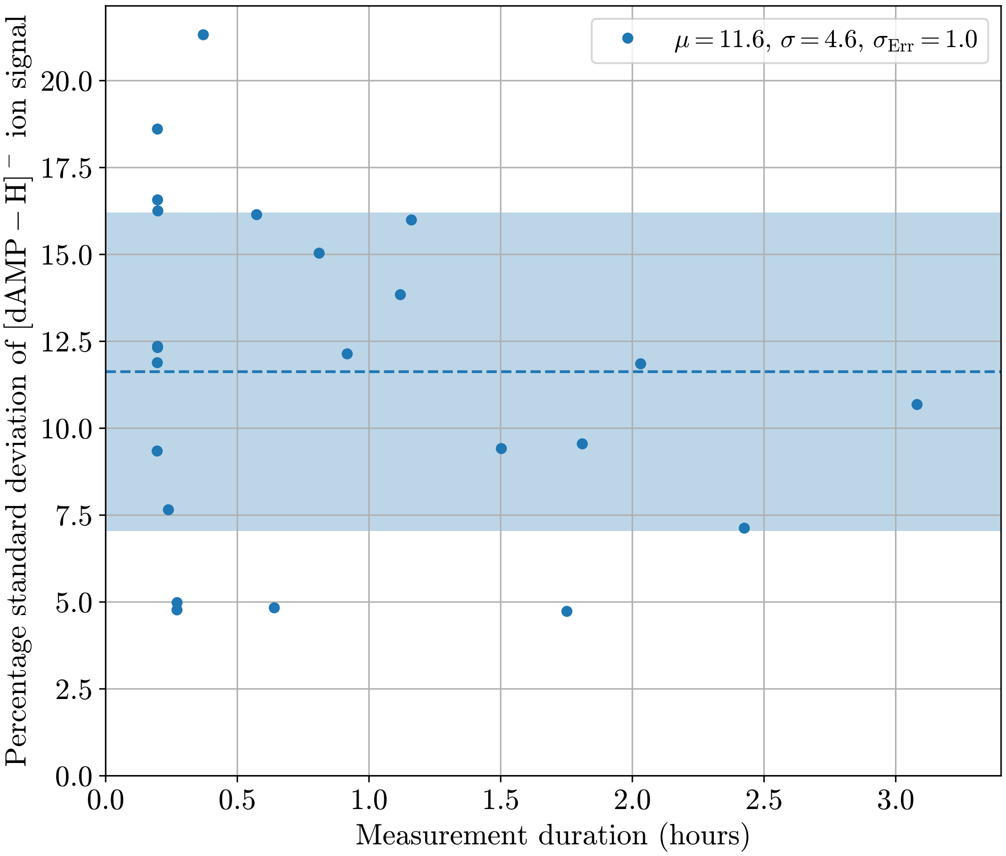

Figure S10: $[\mathrm{dAMP}-\mathrm{H}]^-$ ion signal standard deviation as a percentage of the signal level plotted against the time taken to acquire the measurement. The measurements shown are those used in the spectrum plot as well as the corresponding daily $[\mathrm{dAMP}-\mathrm{H}]^-$ reference measurements. The horizontal dashed line shows the mean of these data with the shaded region depicting the standard deviation.

3.5 Error budget

In this section we list the errors which we account for in the data analysis used to produce Figures 3 and 6 of the main article. These errors are expressed here as a percentage error and are appropriately propagated to calculate error bars.

Table S3: Percentage errors contributing to [dAMP−H]$^-$ spectrum measurement (Figure 3).

Error description	Median	Mean
Statistical error	3.1	4.9
Power normalisation (section 3.1)	5.6	6.2
[dAMP−H]$^-$ decay reference (section 3.2)	3.3	3.5

Table S4: Comparison of the main contributing percentage errors to the uncertainty in the fragment analysis (Figure 6).

Error description	Median	Mean
Relative cross section (Figure 3)	3.9	8.4
Power normalisation (section 3.1)	5.5	6.2
[dAMP−H]$^-$ decay reference (section 3.2)	3.4	3.2
A_F fit error	4.0	4.6
N_0 fluctuations (section 3.4)	12	
Daily laser-ion overlap drifts	14	

The laser-ion overlap drift is estimated using the mean of the empirical factors used for the scaling of these [dAMP−H]$^-$ spectrum measurements described in section 3.2. This error together with the N_0 fluctuation error are taken as fixed values used in the error propagation of each measured point (compared to the other errors listed which are computed from the repeated measurements of the datum being evaluated). We therefore do not quote median and mean values for these errors and instead list the fixed values used for all data points.

References

(1) Spieler, S.; Duong, C. H.; Kaiser, A.; Duensing, F.; Geistlinger, K.; Fischer, M.; Yang, N.; Kumar, S. S.; Johnson, M. A.; Wester, R. Vibrational Predissociation Spectroscopy of Cold Protonated Tryptophan with Different Messenger Tags. *J. Phys. Chem. A* **2018**, *122*, 8037.

(2) Chen, E.; Chen, E.; Sane, N. The Electron Affinities of the Radicals Formed by the Loss of an Aromatic Hydrogen Atom from Adenine, Guanine, Cytosine, Uracil, and Thymine. *Biochemical and Biophysical Research Communications* **1998**, *246*, 228–230.
